# An Investigational RNAi Therapeutic for Tau Lowering

**DOI:** 10.1002/alz.089664

**Published:** 2025-01-09

**Authors:** Jonathan E Farley, Jeffery D Haines, Sean Gannon, Tuyen Nguyen, Roxanne Tymon, Diana Cha, Mark Schlegel, Adam Castoreno, Anna Bisbe, Ivan Zlatev, Jeff Rollins, Andreja Avbersek, Lynn Macdonald, Min Gao, Kirk Brown

**Affiliations:** ^1^ Alnylam Pharmaceuticals, Cambridge, MA USA; ^2^ Regeneron Pharmaceuticals Inc., Tarrytown, NY USA

## Abstract

**Background:**

The hyperphosphorylation, mislocalization, and aggregation of the microtubule associated protein Tau (MAPT) is a driving force in tauopathies, a group of progressive, neurodegenerative disorders. These pathogenic intracellular aggregates, known as neurofibrillary tangles (NFTs), are a hallmark in several diseases such as frontotemporal dementia, progressive supranuclear palsy, and Alzheimer’s Disease. While anti‐Tau immunotherapies emphasize the clearance of extracellular Tau aggregates, they do not address the intracellular accumulation of NFTs.

**Method:**

Here, using our clinically validated C16‐siRNA CNS delivery platform, we have identified potent molecules targeting the *MAPT* gene.

**Result:**

We have identified potent molecules that durably reduce *MAPT* mRNA and Tau protein in both *in vitro* and *in vivo* model systems. Furthermore, in a non‐human primate study, we demonstrated robust, sustained reduction of *MAPT* transcript and tau protein in disease relevant brain tissue that was well‐tolerated through 16‐weeks.

**Conclusion:**

Together, these results suggest that Tau‐lowering via C16‐siRNAi may provide a novel strategy to reduce, and potentially reverse, accumulation of intracellular and extracelluar Tau aggregates. This approach has advanced into in IND‐enabling development.

